# Highly elastic 2D covalent organic framework films of interwoven single crystals

**DOI:** 10.1093/nsr/nwae197

**Published:** 2024-06-08

**Authors:** Tongwen Xu

**Affiliations:** Key Laboratory of Precision and Intelligent Chemistry, Department of Applied Chemistry, School of Chemistry and Materials Science, University of Science and Technology of China, China

Two-dimensional (2D) materials feature angstrom-scale pores and tunable pore chemistry, demonstrating great potential for applications including flexible electronics and separations. The ion/molecule transport in the sub-nanometer-sized pore confinement often reveals phenomena not seen in bulk materials [1]. Yet, the practical application of 2D materials is hindered by their poor mechanical properties.

For instance, 2D covalent organic frameworks (COFs) are constructed by the periodic linkage of organic monomers via covalent bonds [2], which appear as powder materials via the solvent-thermal synthesis method [3] and can be difficult to process into films/membranes. Although 2D COF membranes could be acquired through interfacial polymerization [4], they are polycrystalline and consist of crystals connected via non-covalent interactions, having many grain boundary defects and thus being brittle and fragile. Therefore, it remains an intriguing challenge to concomitantly enhance the mechanical strength and toughness of current 2D COF membranes.

**Figure 1.**
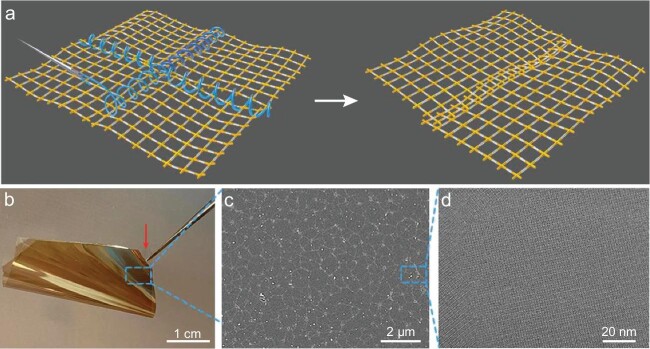
Highly elastic 2D COF films. (a) Strong, tough and elastic COF films of interwoven 2D single crystals. Image Credit: Zhikun Zheng's Group at Sun Yat-sen University. (b) Photograph of COF film deposited onto Nafion film. (c) Scanning electron microscopy image of the continuous COF film of interwoven grain boundaries. (d) Aberration-corrected high-resolution transmission electron microscopy image of the highly crystalline COF film. Reproduced with permission from Ref. [5].

Writing in *Nature*, Zheng *et al*., from Sun Yat-sen University reported a breakthrough in constructing highly crystalline yet elastic COF films by adopting an aliphatic bi-amine as a sacrificial go-between to interweave the grain boundaries of 2D COF single-crystal domains (Fig. 1a and b) [5]. They demonstrate the versatility of this approach with imine-linked 2D COFs due to the high reversibility of imine bonds in water together with high chemical robustness.

Aliphatic bi-amine (i.e. diethylenetriamine) was adopted because of its high reactivity but low reaction constant, as the go-between. Diethylenetriamine promotes the formation of linear chains with aldehyde linkers, facilitating the interweaving of adjacent COF domains at the grain boundary, while the gradual replacement of diethylenetriamine with 5, 10, 15, 20-tetrakis(4-aminophenyl)-21H, 23H-porphyrin generates tough and elastic COF films (Fig. 1c and d). Polyacrylic acid (PAA) acts as the surfactant to regulate the polymerization and crystallization of 2D COFs during interfacial synthesis. The resultant 2D COF films have Young's moduli up to 70 GPa and breaking strengths as high as 80 N/m, competitive with porous ceramic films. Even when fractured, the sliding back and forth of interwoven boundaries prevents the propagation of cracks. The findings reported in this work hold great promise for 2D materials to be utilized in membrane separations. As the authors have shown, when deposited on the Nafion film, the film of 2DCOF-1 stayed intact after folding and crumpling. This would enable the preparation of separation membranes beyond the mixed-matrix membranes or interfacial polymerized COF membranes.

In summary, a sacrificial go-between guided interfacial synthesis approach is developed to introduce interwoven structures to connect single-crystal domains and produce highly strong, tough, and elastic COF films. The sacrificial go-between oriented interwoven grain boundary method will spark research interests in the grain boundary engineering of various polycrystalline materials, and endow the creation of membrane materials with novel functions for emerging separation applications. But there are open questions deserving further investigation, regarding the uniqueness of diethylenetriamine in the film preparation, the role of PAA, and the practical application of elastic 2D COF films.
